# Design of Driving Waveform Based on a Damping Oscillation for Optimizing Red Saturation in Three-Color Electrophoretic Displays

**DOI:** 10.3390/mi12020162

**Published:** 2021-02-07

**Authors:** Zichuan Yi, Weibo Zeng, Simin Ma, Haoqiang Feng, Wenjun Zeng, Shitao Shen, Lingling Shui, Guofu Zhou, Chongfu Zhang

**Affiliations:** 1 College of Electron and Information, University of Electronic Science and Technology of China Zhongshan Institute, Zhongshan 528402, China; yizichuan@zsc.edu.cn (Z.Y.); zengweibo133@126.com (W.Z.); masimin0402@163.com (S.M.); zwjcareer@163.com (W.Z.); Shuill@m.scnu.edu.cn (L.S.); cfzhang@uestc.edu.cn (C.Z.); 2 South China Academy of Advanced Optoelectronics, South China Normal University, Guangzhou 510006, China; shenshitao@m.scnu.edu.cn (S.S.); guofu.zhou@m.scnu.edu.cn (G.Z.)

**Keywords:** electrophoretic displays, driving waveform, damping oscillation, particles separation, red saturation

## Abstract

At present, three-color electrophoretic displays (EPDs) have problems of dim brightness and insufficient color saturation. In this paper, a driving waveform based on a damping oscillation was proposed to optimize the red saturation in three-color EPDs. The optimized driving waveform was composed of an erasing stage, a particles activation stage, a red electrophoretic particles purification stage, and a red display stage. The driving duration was set to 360 ms, 880 ms, 400 ms, and 2400 ms, respectively. The erasing stage was used to erase the current pixel state and refresh to a black state. The particles’ activation stage was set as two cycles, and then refreshed to the black state. The red electrophoretic particles’ purification stage was a damping oscillation driving waveform. The red and black electrophoretic particles were separated by changing the magnitude and polarity of applied electric filed, so that the red electrophoretic particles were purified. The red display stage was a low positive voltage, and red electrophoretic particles were driven to the common electrode to display a red state. The experimental results showed that the maximum red saturation could reach 0.583, which was increased by 27.57% compared with the traditional driving waveform.

## 1. Introduction

As the carrier of human–computer information interaction, a flat panel display is very important in modern life. In recent years, electronic paper displays, which are a new type of display, have occupied a certain share in the display market due to its advantages such as large viewing angle, light in mass, low power consumption, repetitive erasing, and readability under sunlight [1,2,3,4,5]. As a kind of electronic paper display, electrophoretic displays (EPDs) have an excellent performance in the field of device manufacturing, which is expected to become one of the mainstream technologies for next-generation displays [6,7,8]. The charged particles in traditional EPDs are black electrophoretic particles with a positive charge and white electrophoretic particles with a negative charge [9]. They can be driven to the top or bottom of a pixel to display different states by applying an electric field [10,11,12]. However, the black and white particles cannot perfectly express the content of pictures because of the signal color. Hence, a multi-color EPD is urgently needed.

The three-color EPD technology could cover people’s requirements for multi-color electronic papers. In recent years, a three-color EPD has been reported [13]. Electrophoretic particles of three colors were successfully driven in this work, and the EPD can be driven for displaying corresponding colors by different voltage sequences. However, the red and black electrophoretic particles in electrophoretic fluid have the same polarity of charge, and they are driven in the same direction when a driving electric field is applied. The disadvantage of this design is an insufficient color saturation, especially red. Worse still, low display quality can be caused such as ghost image and flicker, which are also problems in traditional EPD displays [14,15,16,17]. In order to improve the display quality of EPDs, a lot of work has been done. The display quality of EPDs has obviously improved by synthetizing new material, manufacturing new devices, and optimizing the driving waveform, etc. [18,19,20]. As optimizing the driving waveform has an obvious improvement in the display quality, researchers have done many works in this aspect. For example, a driving waveform based on a delay response was proposed, and the hysteresis characteristic curve of an EPD was measured [21]. Moreover, Johnson et al. [22] proposed a driving waveform in which a reference state was designed, where the EPD could display the next gray scale more regularly and accurately, and this method could greatly improve the display quality of EPDs. At present, the red saturation of three-color EPDs is insufficient and it could be optimized by improving driving waveforms.

In this paper, an insufficient red saturation of the three-color EPD was improved by designing a new driving waveform. This driving waveform included an addition stage of a damping oscillation; this stage could separate the red and black electrophoretic particles more completely. The saturation is greatly improved when the EPD displayed a red state. At the same time, ghost image and fringe phenomena can be weakened effectively by optimizing other stages of the driving waveform.

## 2. Principle of Electrophoretic Displays (EPDs)

**Figure 1:**
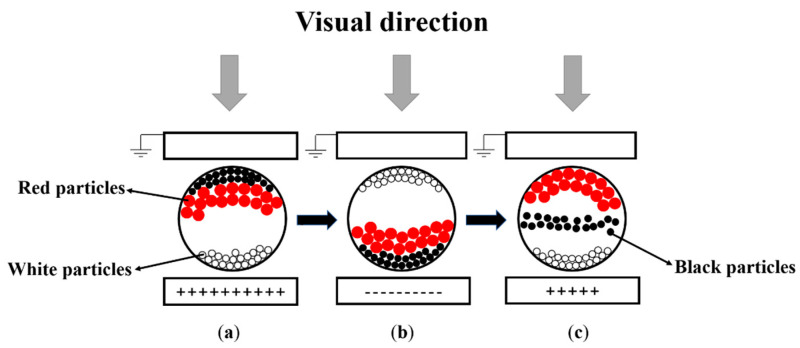
A schematic diagram of a three-color electrophoretic display (EPD). The top is a common electrode, the middle is the microcapsule that contains three-color particles, and the bottom is a pixel electrode. (**a**) When the applied external voltage is a high positive voltage, the pixel is black. (**b**) When the applied external voltage is a negative voltage, the pixel is white. (**c**) When the applied voltage is a low positive voltage, the pixel is red. The particles are in a static state if there is no driving voltage, which is called the bistable state of EPDs.

The structure of a three-color EPD is shown in Figure 1. White electrophoretic particles, black electrophoretic particles, and red electrophoretic particles are wrapped into a microcapsule, and three-color particles have different polarities. The white electrophoretic particles are negatively charged, and the red and black electrophoretic particles are positively charged [13]. Among these particles, the red and black electrophoretic particles are different in charged, mass, and volume. Therefore, they can be successfully separated to display different colors by controlling the magnitude and duration of the driving voltage [23]. As shown in Figure 1a, black electrophoretic particles are driven to the top in a pixel with a high positive polarity voltage. As shown in Figure 1b, white electrophoretic particles are driven to the top in a pixel with a negative polarity voltage. As shown in Figure 1c, red electrophoretic particles are driven to the top in a pixel with a low positive polarity voltage.

The driving waveform refers to a voltage sequence applied to pixels, which seriously affects the display quality of EPDs. A traditional driving waveform is composed of an erasing stage, a particle activation stage, and a target state display stage [24,25]. Changes in each stage can affect the display quality of EPDs. There are many details that need to be improved in the driving waveform for a good display quality such as ghost image, fringe phenomena, flicker, insufficient color saturation, etc. [15,26,27,28]. In the original stage, particles are distributed irregularly in microcapsules. Therefore, it is necessary to rearrange particles to prepare for the next gray scale of EPDs [29]. In this stage, the original state can be driven to a state where electrophoretic particles are regularly arranged to the same state. The migration rate of electrophoretic particles can be decreased when electrophoretic particles remain in the same state for a long time. Therefore, the driving waveform of the particle activation stage is designed. In this stage, pixels are refreshed to black state first, and refreshed to white state immediately. This process can make sure that particles must be activated during the driving process and reach steady state. Next, the target gray scale can be driven by an applied electric field.

Charged particles are subjected to an electric field force in an electric field. As shown in Equation (1):

(1) $$F_{q}=q\times E$$

where *F_q_* is the electric field force; *q* is the electric charge of electrophoretic particles; and *E* is the applied electric field.

Since electrophoretic particles are dispersed in colloidal solution, the movement of electrophoretic particles is also hindered by Stokes force [30]. Its expression is shown in Equation (2):

(2) $$F_{d}=6\pi \mu vr$$

where *F_d_* is the Stokes force; *µ* is the liquid viscosity coefficient; *v* is the motion relative rate between particles and fluids; and *r* is the sphere radius. The combined force of the electric field force and the Stokes force is used as the driving force for the movement of electrophoretic particles. As shown in Equation (3):

(3) $$F=F_{q}-F_{d}=m\frac{dv}{dt}$$

where *F* is the driving force that can drive electrophoretic particles; *m* is the mass of an electrophoretic particle; and $\frac{dv}{dt}$ is the acceleration of electrophoretic particles.

The moving distance of electrophoretic particles in a microcapsule can be calculated according to the integral of speed and time. As shown in Equation (4):

(4) S=∫cdt=∫σEdt

where *S* is the moving distance of electrophoretic particles in a microcapsule; *c* is the moving speed of electrophoretic particles; *σ* is the electrophoretic mobility of electrophoretic particles; and *E* is the applied electric field.

Particles are subjected to random pulse signals in the electric field, as shown in Equation (5):

(5) $$f\left(t\right)=\overset{N}{\underset{n=1}{\sum }}a_{n}\left[u\left(t-nT\right)-u\left(t-nT-\tau \right)\right]$$

where *f(t)* is a random amplitude rectangular pulse signal; *a_n_* is the rectangular pulse amplitude; *t* is the time; *T* is the pulse signal period; and *τ* is the pulse width.

The key step for increasing the red saturation is that red and black electrophoretic particles in microcapsules can be separated completely, which can be completed by designing a damping oscillation driving waveform according to the nature of particles. A damping oscillation refers to the process in which the amplitude of vibration can be gradually decreased over time. As shown in Equations (6) and (7), the characteristic of a damped oscillation is that the amplitude gradually is decreased. Therefore, the intensity of the electric field applied to the pixel gets lower and lower in the driving waveform. Driving black electrophoretic particles requires a high electric field, and driving red electrophoretic particles requires a low electric field. Therefore, the red and black electrophoretic particles can be successfully separated.

(6) $$F_{r}=-Bc$$

(7) $$X\left(t\right)=Ae^{-\delta t}\cos\left(\omega t+\varphi \right)$$

where *F_r_* is the damped force; *B* is the drag coefficient; $Ae^{-\delta t}$ is the amplitude; and *ω* is the angular frequency. In this process, the mean value of time is defined as Equation (8):

(8) $$\bar{X\left(t\right)}=\underset{T\to \infty }{\lim}\frac{1}{2T}\int_{-T}^{T}X\left(t\right)dt$$

## 3. Design of Driving Waveforms

**Figure 2:**
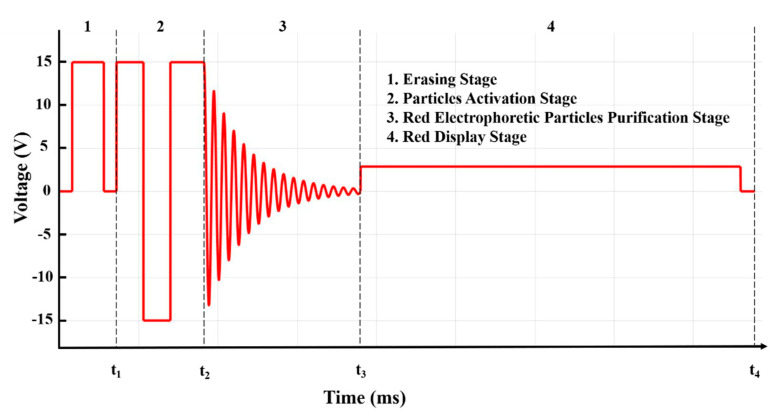
Optimized driving waveform. This is composed of four stages: (**1**) Erasing stage; (**2**) particle activation stage; (**3**) red electrophoretic particles purification stage; and (**4**) red display stage.

To improve the red saturation in three-color EPDs, an optimized driving waveform was designed and divided into four stages: (1) An erasing stage; (2) a particle activation stage; (3) a red electrophoretic particles purification stage, and (4) a red display stage. As shown in Figure 2, the first stage was the erasing stage, which could erase the original pixel state and refresh the white or black state. A positive 15 V could be applied for 200 ms to reset the original state, and the EPD could display a black state. Similarly, a negative 15 V was applied to reset the original state, and the EPD could display a white state. Then, 0 V was applied after 15 V voltage for 80 ms. This duration was set to buffer the electrophoretic particles, and red, black, and white particles were separated at this stage. The second stage was a particle activation stage. The EPDs were driven multiple times to reach the optical limit, which could increase the activity of particles and further eliminate ghost images. At this stage, electrophoretic particles could be driven to move in the colloidal solution with several cycles by an applied voltage. A cycle included a positive 15 V and a negative 15 V, and the duration was 340 ms. Then, the red and black electrophoretic particles were driven to the top of a pixel by a positive 15 V voltage for 200 ms at the end of this stage. The third stage was a red electrophoretic particle purification stage. The purpose of this stage was to separate red and black electrophoretic particles for displaying a red state. Two kinds of particles can be driven at different speeds in microcapsules with the same applied voltage. Hence, the damping oscillation driving waveform had a good effect on separating red and black particles, and the red particles were purified greatly. The fourth stage was a red state display stage, and a low positive voltage was applied during the whole stage.

## 4. Results and Discussion

### 4.1. Construction of Test Platform

**Figure 3:**
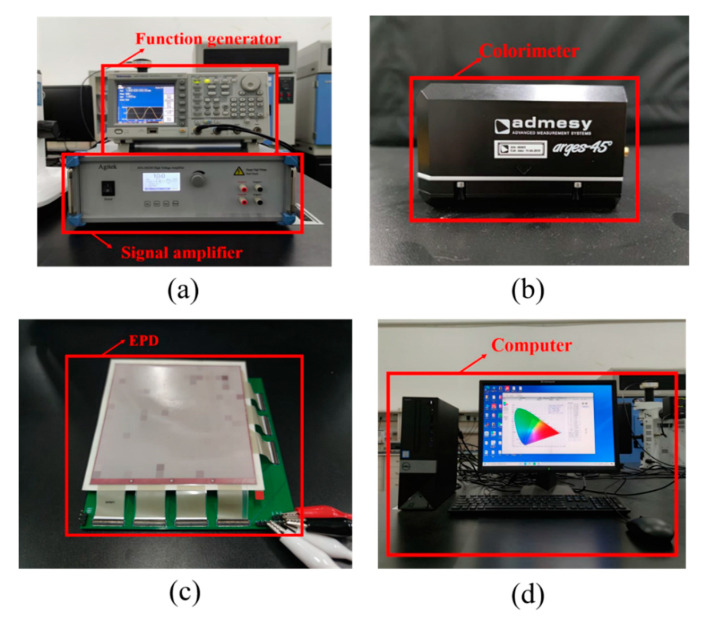
An electrophoretic display (EPD) testing system. (**a**) A function generator and a signal amplifier. (**b**) A colorimeter. (**c**) A microcapsule EPDs. (**d**) A computer.

In this work, the experimental system is shown in Figure 3 and includes a function generator, a signal amplifier, a colorimeter, a microcapsule EPD, and a computer. The microcapsule EPD device was designed by us, and made by foundry (Dalian Longning Technology Co. Ltd., Dalian, China). The driving waveform used in the experiment was first designed by MATLAB (2017, MathWorks, Natick, MA, USA). Subsequently, it was converted to generate a txt format file. Then, the tfw format file was output by Arbexpress (Version 3.4, Tektronix. Inc, Beaverton, OR, USA). Next, the file was burnt into the function generator by a Universal Serial BUS (USB). Finally, the driving waveform was output from the signal amplifier to drive an EPD to display gray scales.

**Figure 4:**
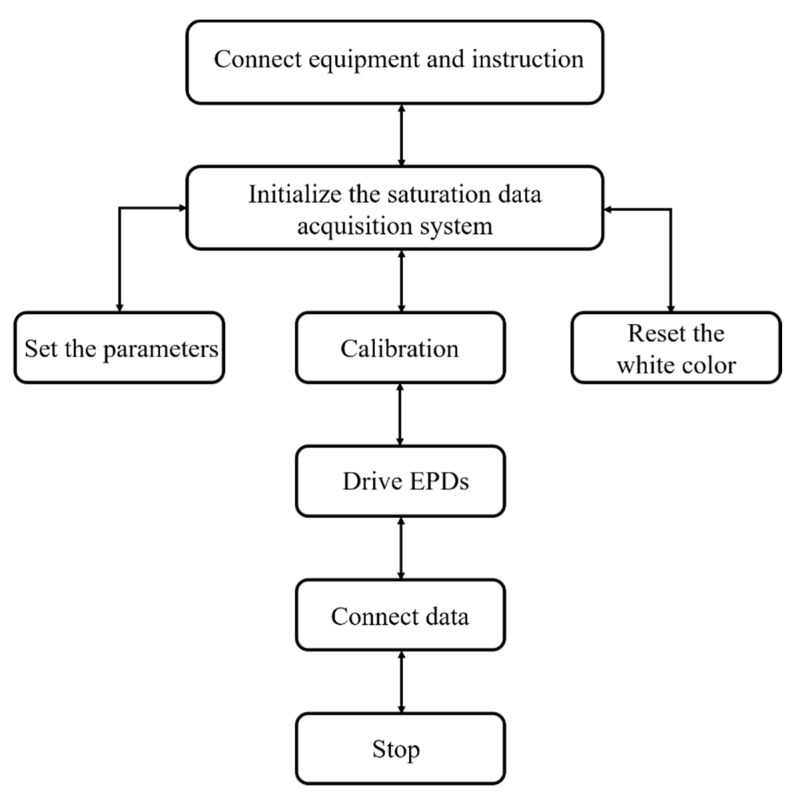
Flow chart of the saturation data acquisition.

The workflow of the entire system is shown in Figure 4. All equipment and instruments were first connected. Then, the saturation data acquisition system was activated, and the relevant parameters were set, which included averaging and trial. Averaging was used to set the time interval for obtaining red saturation data, and the trial was used to set the number of measurements that could appear on the color saturation diagram. Then, the system was effectively calibrated. Finally, the EPD was driven to display relevant colors.

### 4.2. Erasing Stage Optimization

**Figure 5:**
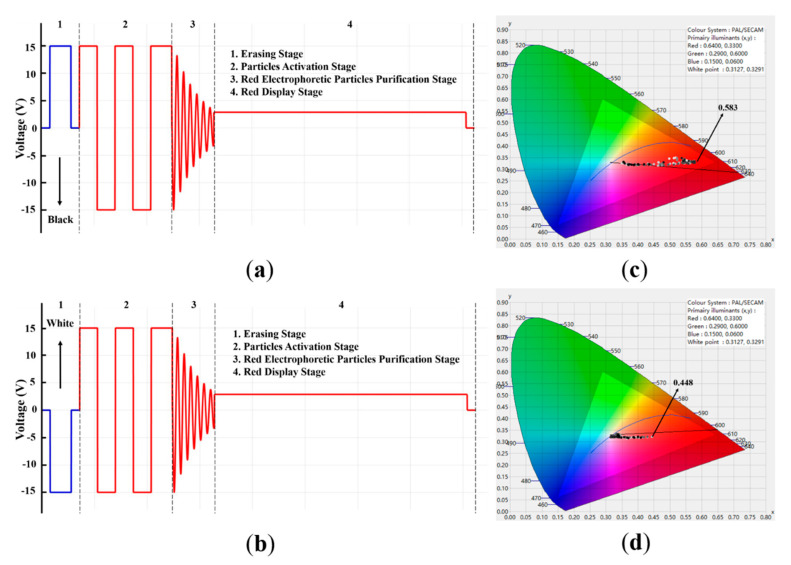
The maximum red saturation when the optimized driving waveform was used in the erasing stage. (**a**) Driving waveform design when it was erased to a black state. (**b**) Driving waveform design when it was erased to a white state. (**c**) The maximum red saturation when the EPD was erased to a black state. (**d**) The maximum red saturation when the EPD was erased to a white state.

Generally, the first stage of the driving waveform is an erasing stage, which is used to erase previous states. In this work, the first stage of the driving waveform was designed to erase the original gray scale and then refresh the EPD to a black state. As shown in Figure 5a, the duration of this stage was set to 360 ms. Compared with the traditional driving waveform, the erasing stage could erase to a white state and the polarity of the applied voltage was the opposite, as shown in Figure 5b. The experimental results showed that when the erasing stage was a black state, the red saturation was 0.583, and the chromaticity diagram is shown in Figure 5c. However, when the erasing stage was a white state, the red saturation was 0.448, and the chromaticity diagram is shown in Figure 5d. This is because when the driving waveform is designed to erase to a white state, white particles were near the common electrode, the red and black electrophoretic particles were at the bottom of a microcapsule. Therefore, it took a lot of time to drive red particles from the bottom to the common electrode in the particle activation and display red color stages.

### 4.3. Particle Activation Stage Optimization

**Figure 6:**
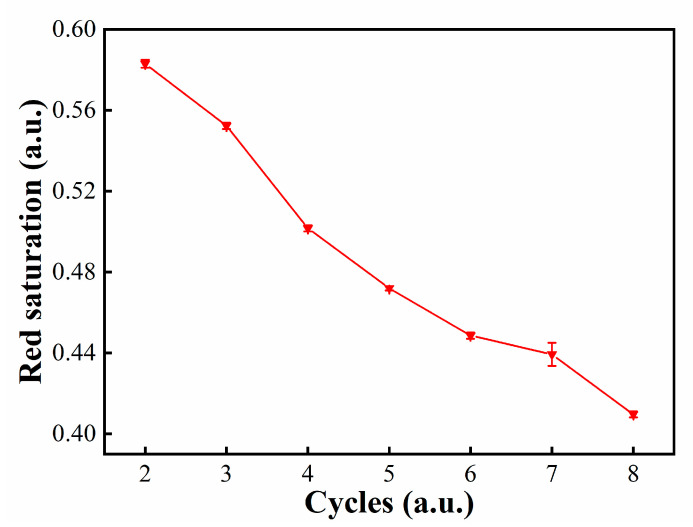
The relationship between cycles and the maximum red saturation.

According to the traditional driving waveform, the particle activation stage has an important contribution to reducing ghost images. At this stage, different cycles were tested. The duration of a cycle was designed to be 340 ms, and the duration for resetting to the black state was 200 ms. The maximum red saturation with different cycles are shown in Figure 6. The experimental results showed that the maximum red saturation could reach 0.583 when the stage was two cycles. As the cycle was increased, the maximum red saturation was gradually decreased. This is because electrophoretic particles could not return to their original position due to the resistance of the colloidal solution when the EPD was driven to the black state or the white state. After multiple cycles, the distance was increased so that white electrophoretic particles could not be driven to the bottom in a microcapsule.

### 4.4. Waveform Design of the Damping Oscillation

**Figure 7:**
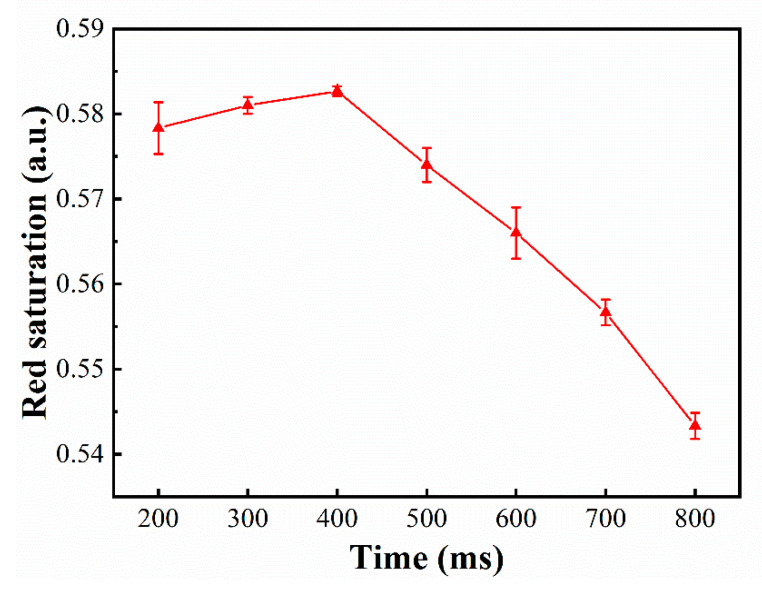
The relationship between the damping oscillation duration and the maximum red saturation of the target state.

Since the red and black electrophoretic particles have the same polarity charge, it was unrealistic to separate them by simply changing the driving voltage polarity. However, the nature of red and black electrophoretic particles is different. A damping oscillation driving waveform can not only change the polarity, but also change the magnitude of the electric field. Hence, the red and black electrophoretic particles can be separated by the damping oscillation driving waveform. As shown in Figure 7, we tested the effect of different damping oscillation durations on the red saturation. It could be seen that the maximum red saturation could be obtained when a short duration was designed for the damping oscillation, and the maximum red saturation could be gradually improved when the duration of the damping oscillation was increased to 400 ms. When the duration exceeded 400 ms, the maximum red saturation gradually became worse. This was because the damping oscillation duration was too short, so the red and black electrophoretic particles were not completely separated. Then, the black particles were under red particles when the target state was red so that the red saturation was low. In addition, when the damping oscillation duration was too long, the red and black electrophoretic particles were mixed again. Hence, the red saturation was also low.

### 4.5. Red Display Stage Optimization

**Figure 8:**
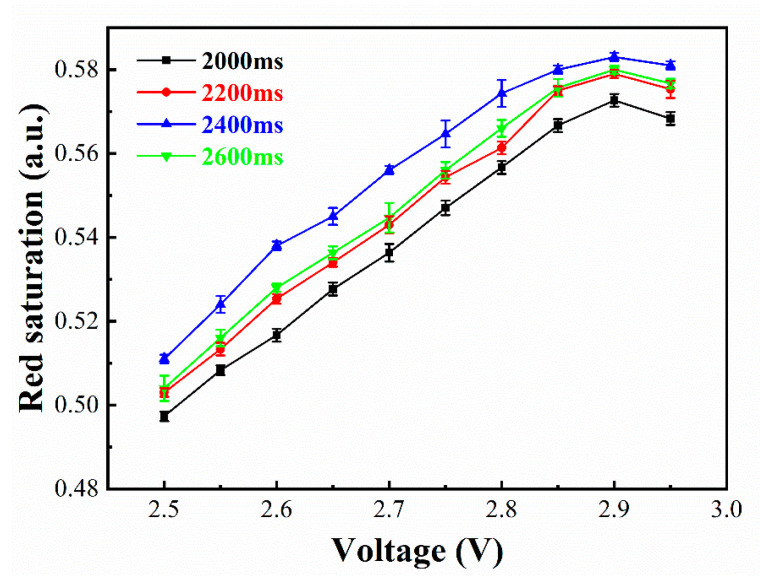
The relationship between the maximum red saturation of the target state and the driving voltage with different durations.

In order to obtain a high red saturation, the driving waveform was optimized in two aspects: driving voltage and duration. As shown in Figure 8, the influence of different driving voltages on the red saturation was verified by designing different durations. The experimental results showed that the maximum red saturation values were different with different durations. The maximum red saturation was gradually improved when the duration was increased from 2000 ms to 2400 ms and the maximum red saturation showed a downward trend when it exceeded 2400 ms. This is because a long duration would drive black electrophoretic particles toward the common electrode. In addition, the red saturation showed an upward trend when the driving voltage was increased from 2.5 V to 2.9 V, and the red saturation was gradually decreased when it exceeded 2.9 V. Comprehensively, the optimal parameters were the driving voltage of 2.9 V and the duration of 2400 ms.

**Figure 9:**
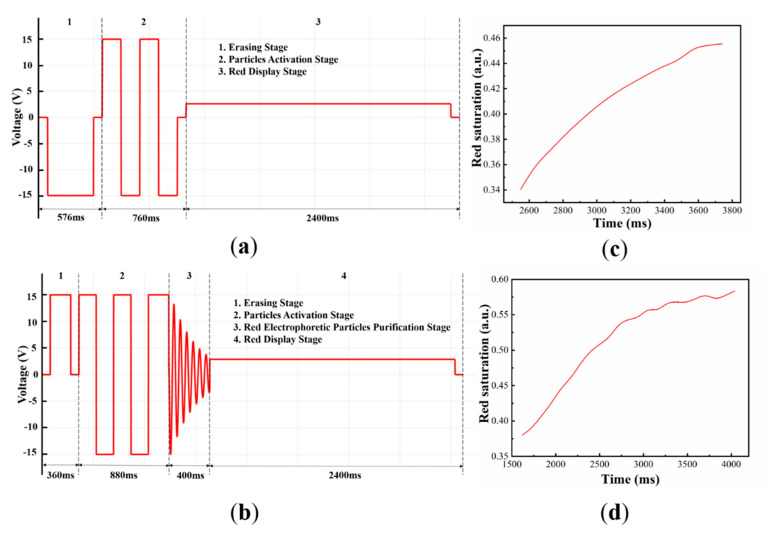
(**a**) The traditional waveform which was composed of three stages: (1) The erasing stage, (2) particle activation stage, (3) target state display stage. The driving waveform duration of three stages was set to 576 ms, 760 ms, and 2400 ms, respectively [13]. (**b**) The optimized driving waveform was composed of four stages: (1) The erasing stage, (2) particle activation stage, (3) red electrophoretic particles purification stage, and (4) red display stage. The driving waveform duration of the four stages were set to 360 ms, 880 ms, 400 ms, and 2400 ms, respectively. (**c**) The relationship between the red saturation and red display stage based on the traditional driving waveform. (**d**) The relationship between the red saturation and red display stage based on the optimized driving waveform.

We tested the maximum red saturation based on the traditional driving waveform and the optimized driving waveform, respectively. As shown in Figure 9, the experimental results showed that the maximum red saturation was increased from 0.457 to 0.583, therefore the red saturation increased by 27.57%.

## 5. Conclusions

In this paper, a driving waveform that could optimize red saturation was proposed for three-color EPDs. The damping oscillation driving waveform could separate red and black electrophoretic particles very well. Compared with traditional driving waveforms, the red saturation of the optimized driving waveform could be effectively improved by 0.126. At the same time, the ghost image could be reduced and the steady state of particles could be improved. The concept of the damping oscillation can provide effective design ideas for the design of driving waveforms for color EPDs, which can provide a better and more comfortable visual experience for users.

## Data Availability

Data is contained within the article.
